# Supplementary material for: Twisting DNA by salt
Source: Nucleic Acids Res. 2022 May 30;50(10):5726–38. doi: 10.1093/nar/gkac445 (PMC9177979; doi:10.1093/nar/gkac445)
Supplement: gkac445_Supplemental_File [file gkac445_supplemental_file.pdf]

Supporting Information: Twisting DNA by Salt

Sergio Cruz-León,[†,¶] Willem Vanderlinden,[‡,¶] Peter Müller,[‡] Tobias Forster,[‡] Georgina Staudt,[‡] Yi-Yun Lin,[‡] Jan Lipfert,[*,‡] and Nadine Schwierz[*,†]

†*Department of Theoretical Biophysics, Max Planck Institute of Biophysics, Max-von-Laue-Str. 3 60438 Frankfurt am Main, Germany*

‡*Department of Physics and Center for Nanoscience (CeNS), LMU Munich, Amalienstr. 54, 80799 Munich, Germany*

¶*These authors contributed equally.*

E-mail: jan.lipfert@lmu.de; nadine.schwierz@biophys.mpg.de

Supplementary methods

Molecular dynamics simulations

We performed independent simulations of the dsDNA for each ion type and salt concentration. Simulations included periodic boundary conditions and electrostatic interactions were treated using particle-mesh Ewald summation[S1] with tin foil boundary conditions. We used a 2 fs time step with constraints on the hydrogen bond atoms using the LINCS algorithm.[S2] The long-range electrostatic interactions were treated with cubic interpolation and a Fourier space grid of 0.12 nm. Lennard Jones interactions and close Coulomb real space interactions were cut-off at 1.2 nm. Errors from the truncation of LJ interactions were accounted for by long-range dispersion correction for energy and pressure.

Simulation protocol: The dsDNA structure was placed in an orthorhombic dodecahedral box, assuring a minimal distance of 1.5 nm to the edge. The simulation box was filled with 71011 TIP3P water molecules.[S3] Subsequently, the number of ions required for a given concentration was estimated based on the number of water molecules in the simulation box plus the ions necessary to neutralize the charge of the DNA (see Tables S1 and S2). Water molecules were randomly replaced by ions to obtain a neutral system with the desired salt bulk concentration.

A pre-equilibration protocol, consisting of energy minimization, NVT equilibration and NPT equilibration, was completed prior to production runs. During the pre-equilibration process, the heavy atoms of the nucleic acids were constraint with a soft harmonic potential with a force constant of 1000 kJ/(mol nm^2) to allow the equilibration of the solvent around the nucleic acid. Energy minimization used the steepest descent algorithm with a maximum of 50000 steps. Later, we employed 1 ns NVT and 1 ns NPT simulations to further equilibrate the system while keeping the positions restraints.

Finally, unrestrained runs were performed in the NPT ensemble. Trajectories were 3 μs long for monovalent cations and 5 μs long for divalent cations. NPT simulations used

the isotropic Parrinello-Rahman barostat[S4] and the velocity rescaling thermostat with a stochastic term.[S5]

Analysis of trajectories: Helical structural parameters were analyzed with the broadly used software tools `3DNA`[S6] and `do_x3dna`,[S7] complemented with in-house scripts. Particularly, we obtained the helical-twist, helical-rise, the base-pair (bp-)twist, and bp-rise, the radius r, the sugar pucker P. r was defined as the mean distance of the phosphorous atoms to the helical axis. Finally, the helical crookedness β, previously introduced,[S8] is measured from the ratio between the sum of helical rises h and the sum of bp-rises d ($\cos\beta = \mathrm{h/d}$). For the analysis of the helical parameters, the last three bases at each end were not considered. The reported error values correspond to the standard error of the mean (SEM) after block averaging. The cations distributions were analyzed using the `canion` tool of the software `Curves+`,[S9] which calculates the concentration profiles in untwisted helicoidal coordinates, allowing a detailed view of the interaction of the ions at specific volumes in the DNA. The final bulk concentration was obtained from `canion` (see Table S3). Complementary, gromaρs was used to obtain the three-dimensional distribution of the ions,[S10] and the results were visualized with `pymol`.[S11]

Definitions of the twist in the simulations

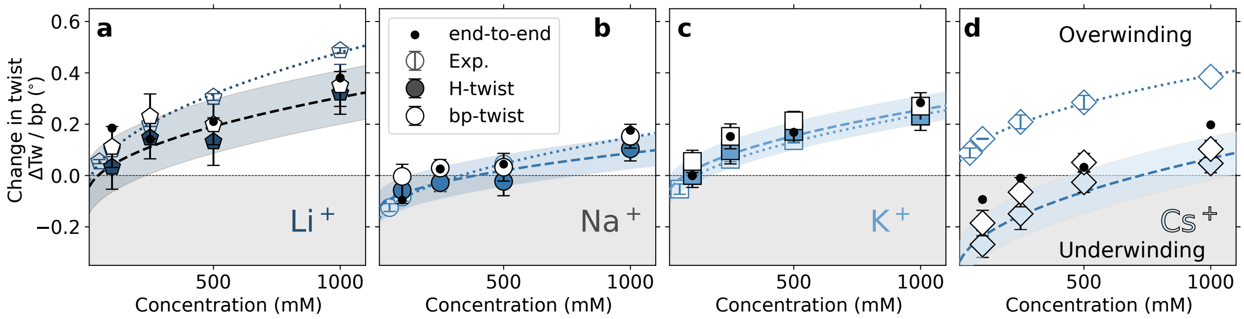

Figure S1: Comparison of ΔTw obtained from three different definitions: (i) the sum of the bp-twist (white symbols), (ii) the sum of the helical-twist (filled symbols), and (iii) the end-to-end twist definition as described by Kriegel et al.[S12] (points). Empty symbols are the experimental results. The dotted lines are the fitting of the experimental results to the square root of the cation concentration. To compare the different methods, the dashed line and the transparent area corresponding to the fitting of the helical-twist to the square root of concentration and its error are plotted.

To compare the structural changes of the DNA with the changes observed in experiments, we calculated the change in twist using three different definitions, as previously discussed:[S12] (i) the sum of the bp-twist, that is, the sum of local twist of a dinucleotide step. (ii) the sum of the helical-twist. It corresponds to the sum of the rotation angles about the helical axis that brings successive base pairs into coincidence. (iii) the end-to-end twist definition described by Kriegel et al..[S12] In this definition, a base-fixed coordinate reference frame[S13] is assigned to both ends of the helix using quaternions averaging[S14] over three base pairs at each end. The total twist corresponds to the rotation of the X-Y plane of the reference frames.[S12] For each definition, we obtained the change in the twist for every recorded snapshot.

The three methods yielded similar relative changes for the change in twist ΔTw, as shown in Figure S1. In the following, we report the end-to-end twist because it has the property of being invariant with respect to initial constant rotation offsets about the z-axis,[S12] and it reproduces the closest the experimental setup where the initial torsional offset of the beads is not known. For a detailed discussion, see Ref.[S12]

Influence of duplex length on the calculated change in twist

Furthermore, recent experimental[S15] and computational evidence[S16,S17] have shown that the sequence strongly influences the equilibrium conformation of DNA. In our magnetic tweezers experiments, one would expect this effect to average out as measurements included a mixed sequence with 7900 bps. In contrast, for simulations of 33 bp it is not clear, a priori, how the sequence and length of the simulated helix may affect the relative changes in twist. To assess this effect, we computed ΔTw for different duplex lengths. Hereby, we subsequently removed the last bp at each end of the simulated helix (Figure S2). Our results show similar changes up to 15 bp (see Figure S2), which indicates that sequence effects average out with the 33 bp sequence used in the simulations.

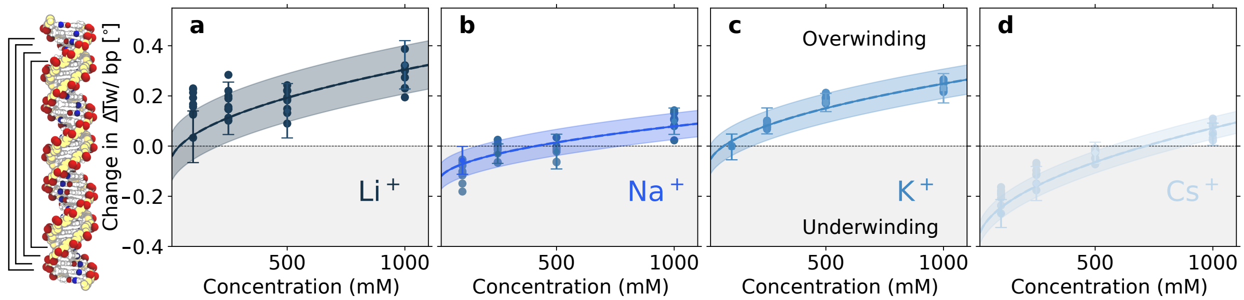

Figure S2: Dependence of ΔTw on the duplex length. ΔTw was calculated for the central section of the helix of different lengths between 15 and 27 bps by removing the bases at each end (scattered points). For comparison, the fit (continuous line) for the change in twist for 27 bps and its error (the transparent area) are shown.

Supplementary experimental results

Fit of the experimental change in twist with concentration

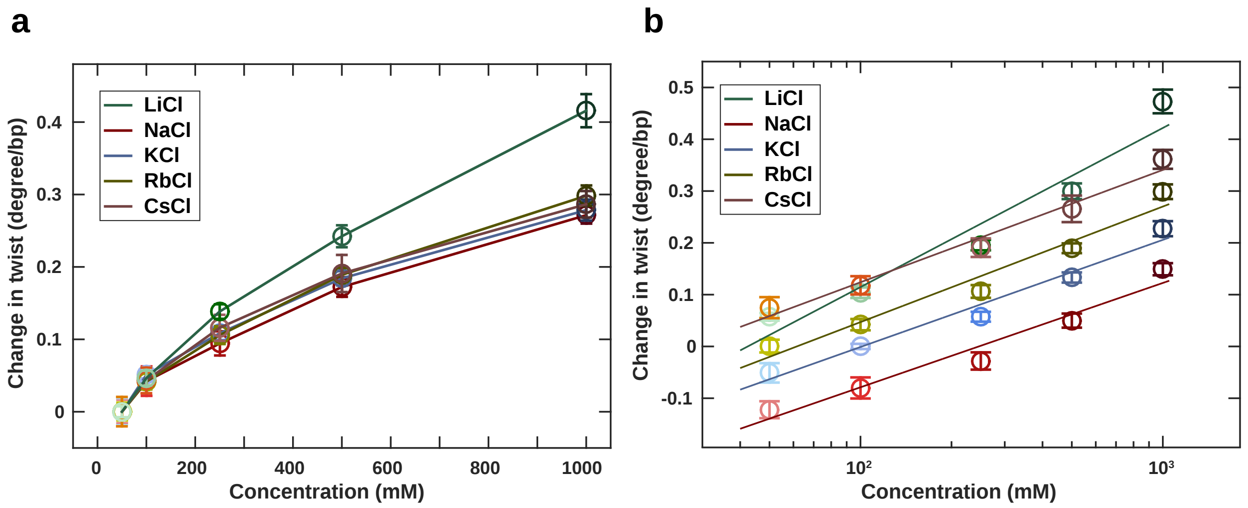

Figure S3: Change in DNA twist for monovalent cations as function of concentration. Symbols and error bars are the mean and standard deviations from at least eight independent molecules (except for Cs^{+}).(a) Data are shifted to the corresponding 50 mM data point and shown in a linear-linear plot. (b) Data on a log-linear plot with respect to 100 mM KCl. Lines in panel (b) are the fit to the log of the ion concentration model with a reduced $\chi^2 = 29.1$.

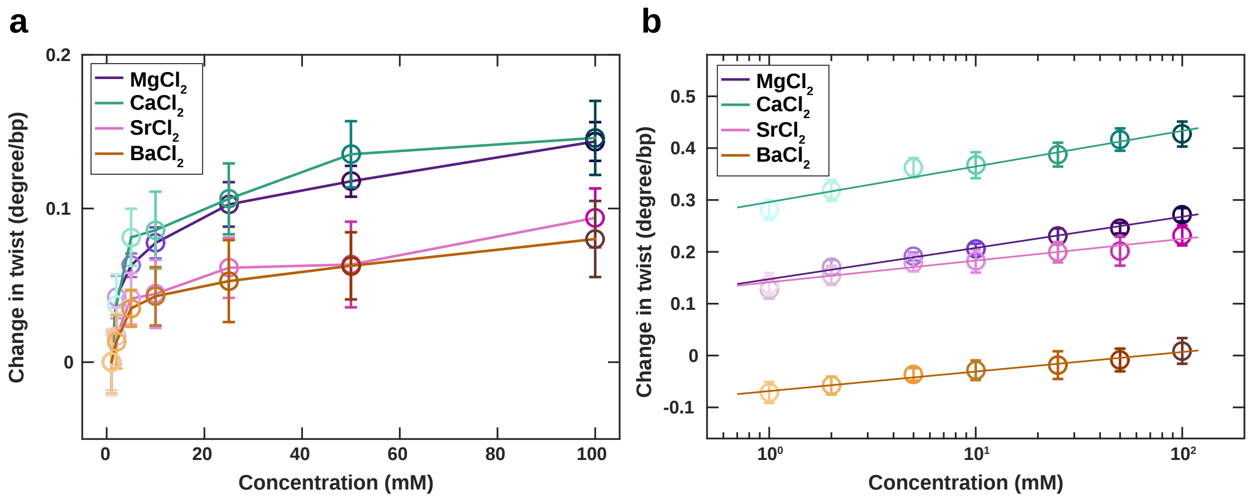

Figure S4: Change in DNA twist with ion type and concentration for divalent ions. Symbols and error bars are the mean and standard deviations from at least seven independent molecules. (a) Data shifted to the corresponding 1 mM data point and shown in a linear-linear plot. (b) Data plotted in a log-linear plot with respect to 100 mM KCl. Lines are the fit to the log of the ion concentration model with a reduced $\chi^2 = 0.84$.

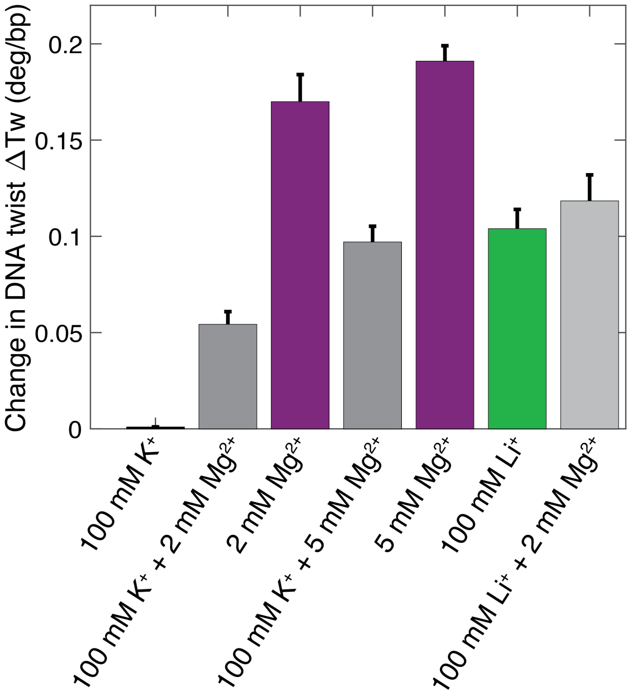

Figure S5: DNA twist in the presence of multiple metal ions. Changes in DNA twist, relative to 100 mM KCl, for mixed ion conditions. The single ion conditions for 100 mM KCl, 100 mM LiCl, and 2 and 5 mM $MgCl_2$ are reproduced from Tables S5 and S7 and are shown for comparison. Combining 2 or 5 mM $MgCl_2$ with 100 mM KCl gives a DNA twist intermediate between the respective single ion conditions. Similarly, combining 2 mM $MgCl_2$ and 100 mM LiCl gives a response that is intermediate between the 100 mM LiCl or 2 mM $MgCl_2$ only conditions. This intermediate response is consistent with a view where in the presence of multiple metal ions the different species "compete" for occupancy in the ion atmosphere around the nucleic acid[S18,S19] and where the resulting concentrations of ions in close proximity to the DNA is intermediate between the corresponding single ion conditions. Comparison to ion counting experiments[S18] suggests that the effect on DNA twist is at least approximately proportional to fraction of charge neutralized by the respective ion species in the ion atmosphere. The DNA twist for multiple ions in solution was determined similar to the measurements for single ion species reported in the main text. Rotation curves where first recorded for 100 mM KCl as the reference conditions. Subsequently, rotation curves were recorded in the mixed buffer conditions and the change in DNA twist was determined from the centers of the rotation curves. Bars and error bars are the mean and standard deviation from measurements with 18-20 independent molecules for the mixed ion conditions.

Supplementary simulation results

Changes in major and minor grooves

As shown in Figure S6, the width of the minor groove changes with the ion type and concentration. Li^+ compacts the minor groove the most, followed by K^+, Na^+, and Cs^+. In contrast, the major groove width is essentially unaffected by Li^+ and Na^+, but it is compressed by K^+ and Cs^+ (Figure S6), reflecting the accumulation of the ions in the grooves as discussed in the main text.

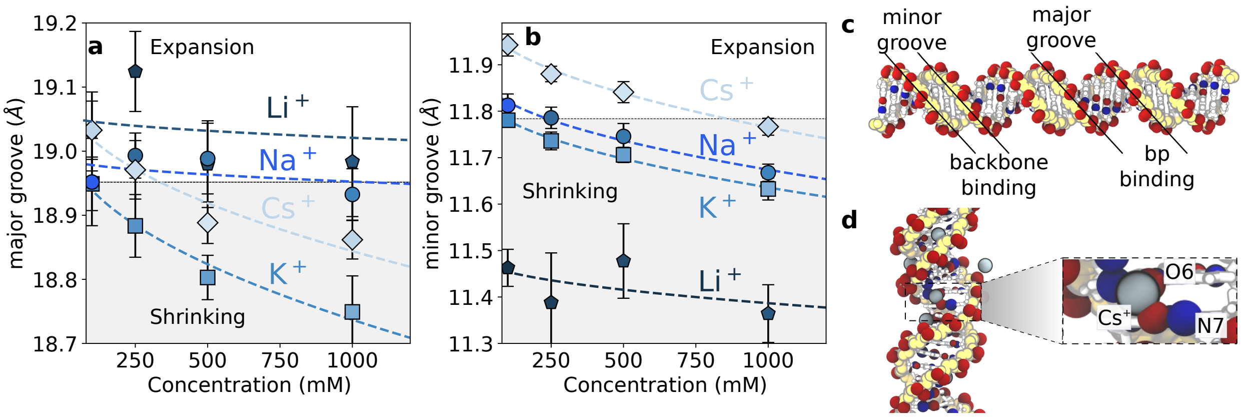

Figure S6: (a,b) Changes in the minor groove and the major groove width depending on ion type and concentration. Error bars correspond to the SEM. (c) Snapshot of the simulated DNA structure indicating the binding volumes. (d) Snapshot of s segment of DNA with a detail of Cs^+ bound at the major groove (atoms N7 and O6) pulling two consecutive bases together.

Helical parameters as function of ion type and concentration

Similar to ΔTw, we defined the change for all the helical properties relative to 100 mM of KCl.

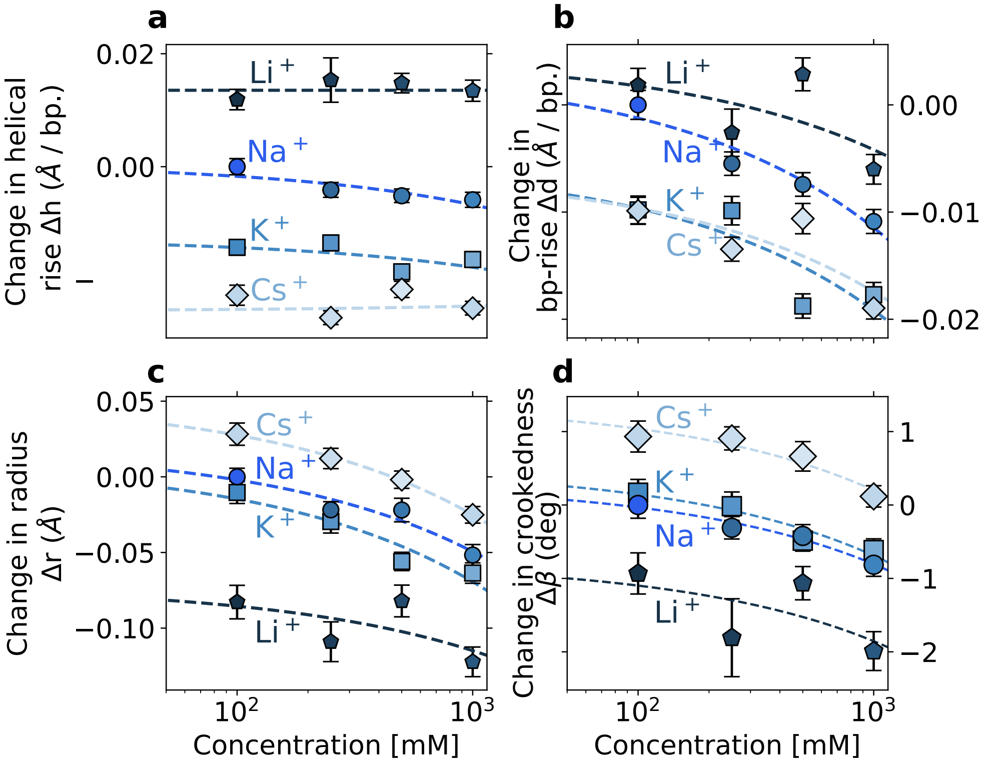

Figure S7: Changes in DNA helical properties as function of monovalent ion type and concentration. (a-d) Changes in the helical rise Δh, the bp-rise Δd, the helical radius Δr, and crookedness $\Delta\beta$, respectively, with respect to 100 mM of KCl. Dashed lines correspond to fittings proportional to the square root of the cation concentration.

Binding for K^+ and Na^+ as a function of DNA sequence

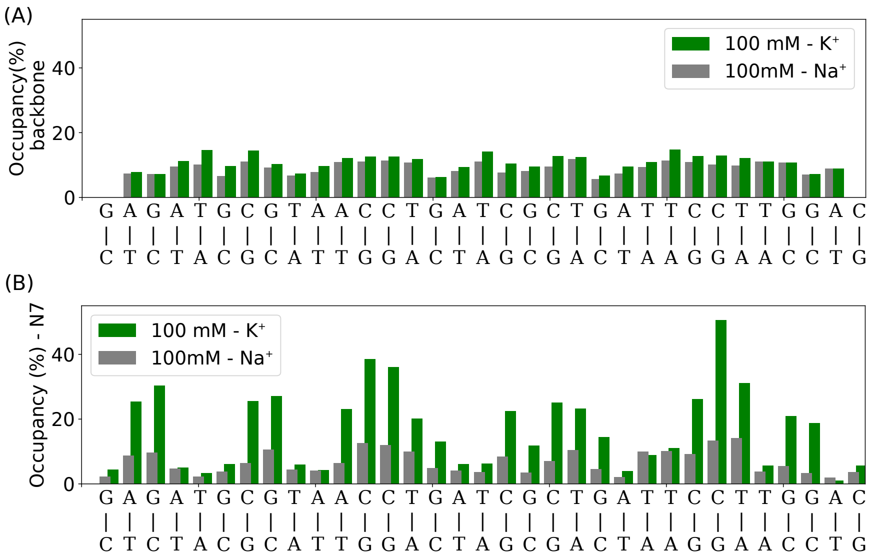

Figure S8: Time averaged occupancy of the main binding sites at the backbone (A) and the nucleobase (atoms N7) of the DNA as a function of the sequence (B). The comparison shows 100 mM of KCl and 100 mM of NaCl. A binding site was considered occupied when a cation was within a cutoff of 3.5 Å of the binding site. In agreement with X-ray experiments,[S20] our simulations show a preferential binding of K^+ to the G-tracks of the helix.

DNA backbone sub-states in the simulations

As a possible source of change in twist, we evaluate the DNA backbone sub-states, which have been related to changes in the local twist.[S17] In particular, we investigated the sub-states BI and BII defined from the torsion angles ε and ζ as BI ($\varepsilon - \zeta < 0$) and BII ($\varepsilon - \zeta > 0$). However, we did not find significant changes in the population of BI/BII sub-states (Figure S9), in agreement with recent results.[S12]

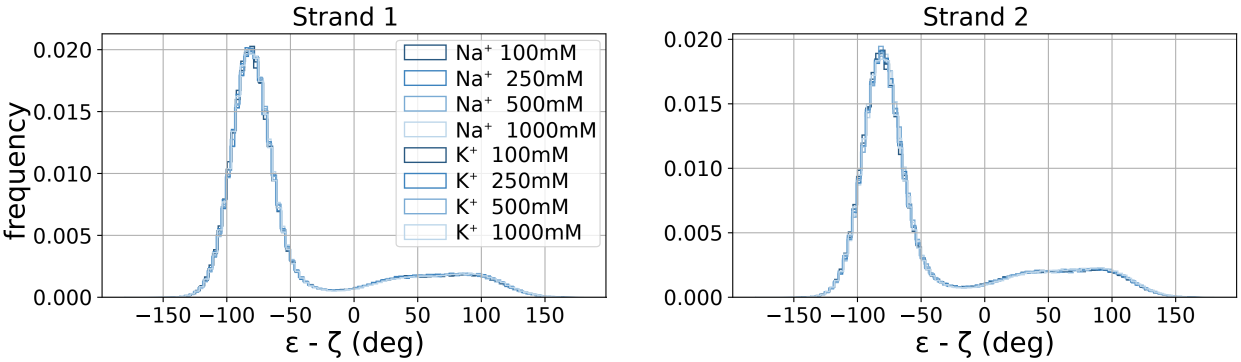

Figure S9: Population of the DNA backbone sub-states BI ($\varepsilon - \zeta < 0$) and BII ($\varepsilon - \zeta > 0$).

Changes in twist for divalent cations

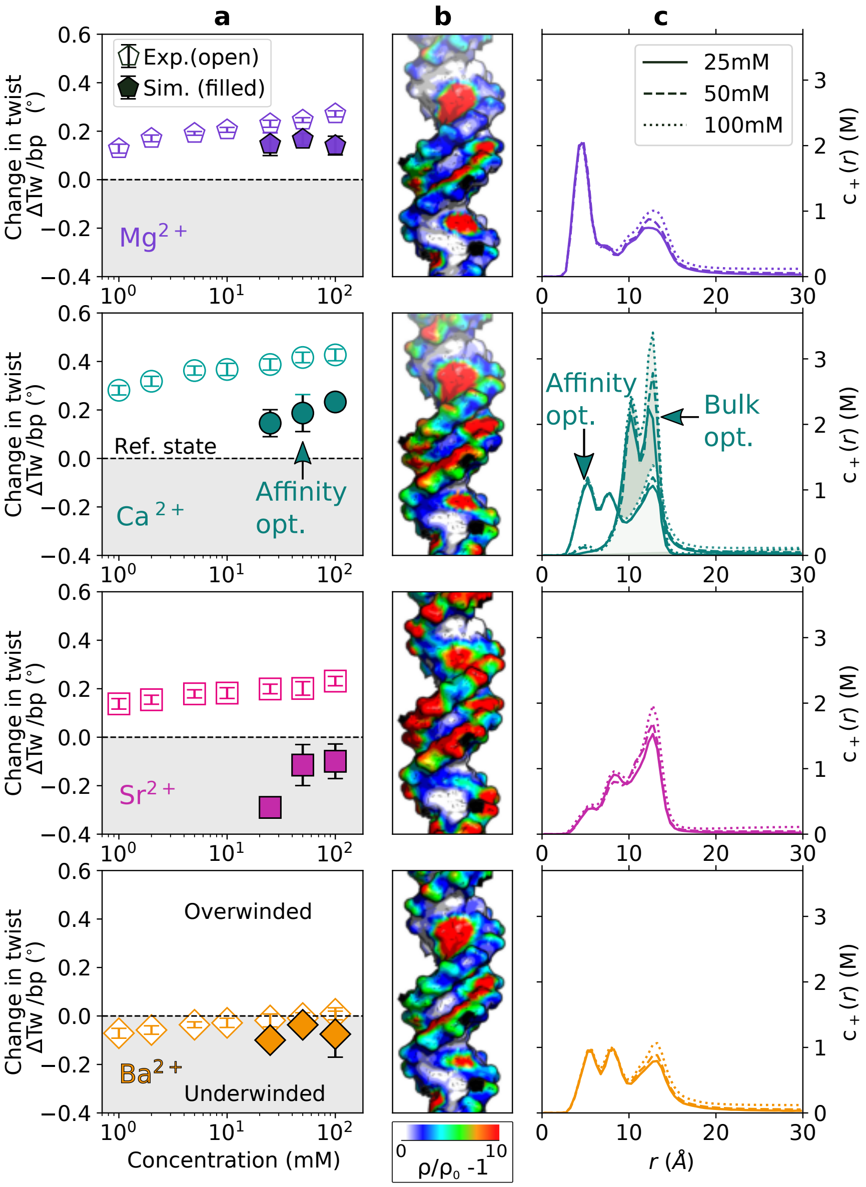

Figure S10: Change in twist for divalent cations. (a) Quantitative comparison of DNA twist obtained from MT experiments (open symbols) and MD simulations (filled symbols) as a function of the ion concentration. (b) Three-dimensional ion distributions. (c) Radial concentration profiles for 25 - 100 mM bulk salt concentration. For Ca^{2+}, the concentration profiles for the two force fields[S21,S22] used in this work are shown (see main text for further details).

Helical properties for divalent cations

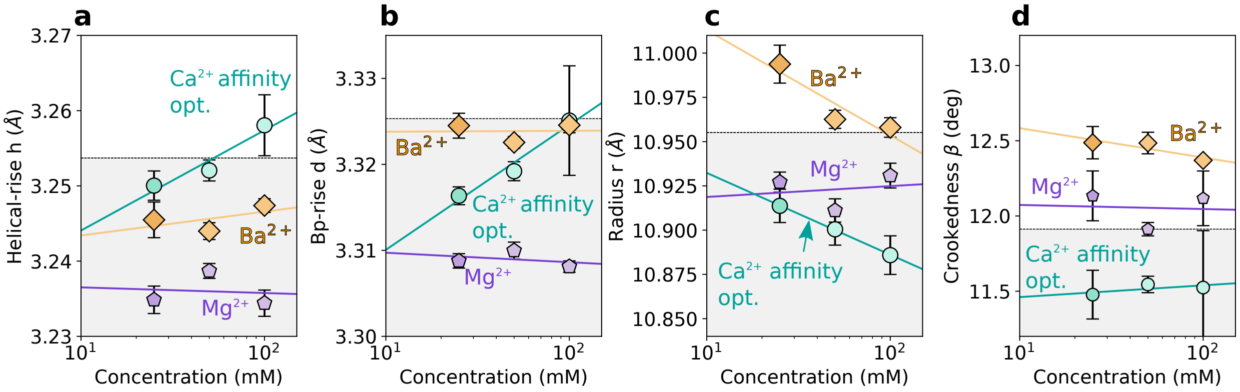

Figure S11: Changes of the characteristic DNA properties for different divalent cations: helical rise h, bp-rise d, radius r, and crookedness β for Mg^{2+}, Ca^{2+}, and Ba^{2+}. All results are relative to 100 mM KCl. For Ca^{2+}, the data are for the affinity optimized parameter set from Ref.[S22] In all cases, the lines are a fit proportional to the natural logarithm of the concentration.

Change in twist using TIP3P and SPC/E water models

We performed a set of four control simulations using the SPC/E[S23] water model to observe the effect of the water model on the change of twist. Namely: 100, 500, and 1000 mM of KCl and 1000 mM of NaCl. The SPC/E simulations started from the last frame of the corresponding TIP3P simulation and each calculation was 200 ns long. All other parameters were identical to the TIP3P simulations. For SPC/E simulations, we performed the twisting analysis and obtained the relative change in a twist (Figure S12).

The SPC/E water model reproduces the increase of twist with concentration for K^+ (Figure S12A,B) and the relative larger twist of k_BT relative to Na^+ at 1000 mM qualitatively. However, the change in twist predicted by SPC/E is larger than the change predicted by TIP3P. Even though SPC/E water reproduces the physical properties of water better compared to TIP3P, the agreement with the experimental DNA twist is worse for SPC/E. Note, that the less convincing agreement for DNA twist in the SPC/E simulations is likely caused by the fact that the ion force fields were optimized in combination with TIP3P water (and not SPC/E). To improve the performance in SPC/E, a more systematic comparison with several ion force fields would be required which is beyond the scope of our current work.

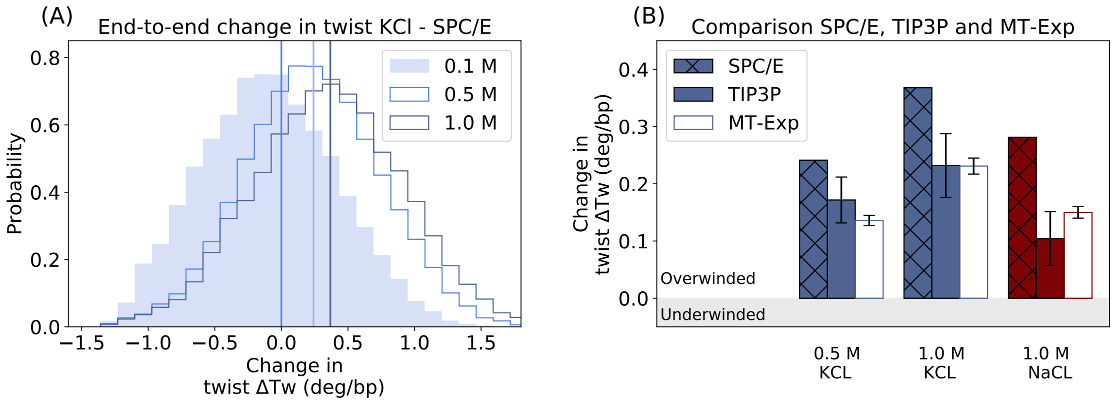

Figure S12: Comparison of the changes in twist obtained for the water models SPC/E and TIP3P with MT-experiments. (A) Histograms of the change in twist for the SPC/E model. Results correspond to 0.1 (reference), 0.5, and 1.0 M of KCl in SPC/E. (B) Comparison of the relative changes in twist ΔTw obtained with SPC/E, TIP3P and MT-experiments for Na^+ and K^+. Note that the changes in twist ΔTw were estimated using the reference state with a given water model, i.e., the reference state for SPC/E is the simulation of 0.1 M of KCl with SPC/E water.

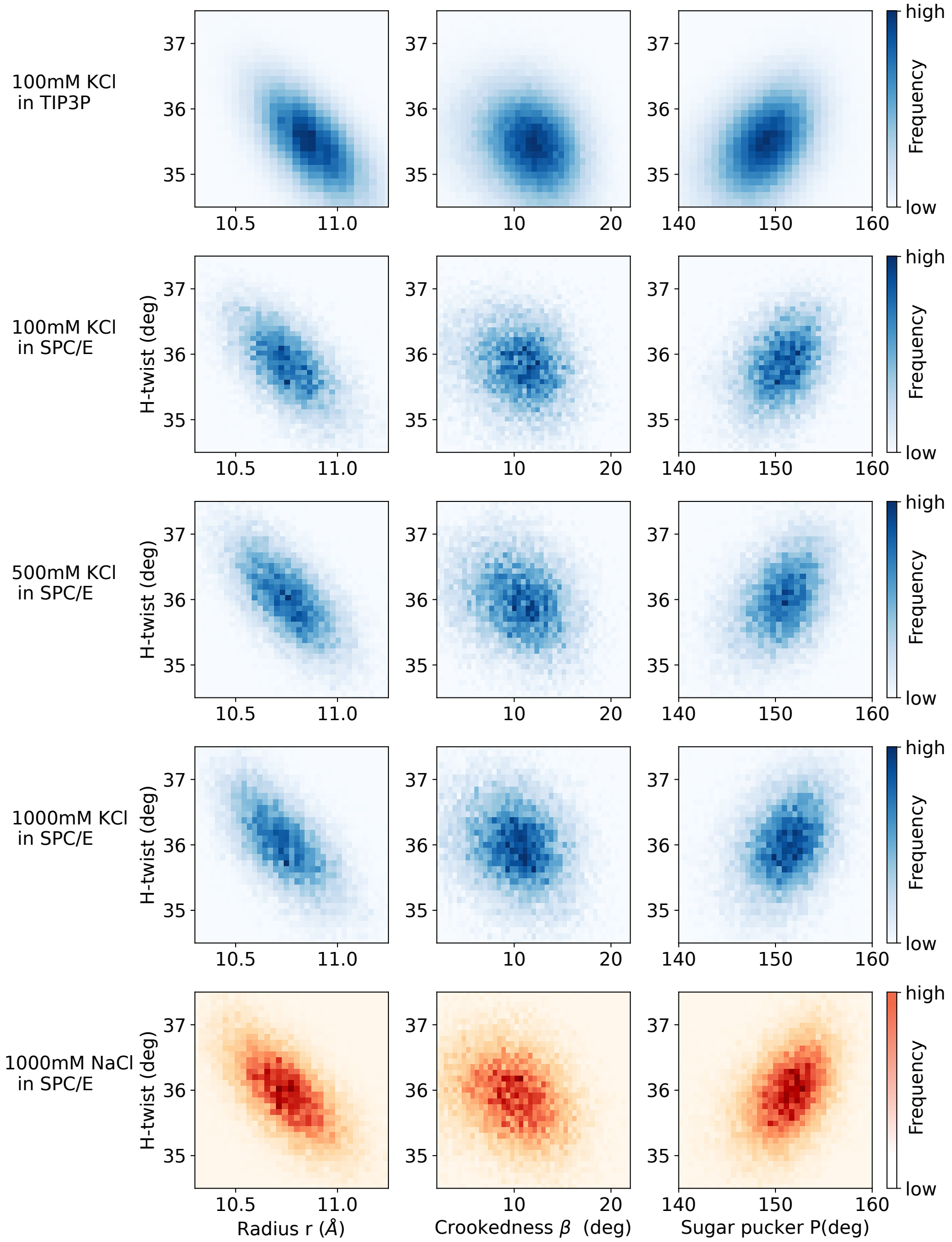

Figure S13: Correlations between helical properties obtained for TIP3P and SPC/E water models. The two-dimensional histograms were calculated for the central 27 bp and for the total simulation time, i.e., 3000 ns for TIP3P and 200 ns for SPC/E. Plots in blue and orange are for K^+ and Na^+, respectively. The SPC/E water model exhibits similar trends for the correlation between helical properties as the TIP3P model.

Supplementary Tables

Table S1: Number of added monovalent cations and anions for the simulations.

Concentration (mM)	number of water molecules	cations	anions
100	70691	192	128
250	70309	383	319
500	69669	703	639
1000	68389	1343	1279

Table S2: Number of added divalent cations and anions for the simulations.

Concentration (mM)	number of water molecules	cations	anions
25	70883	64	64
50	70787	96	128
100	70499	192	320

Table S3: Bulk concentrations obtained in the simulations of mono- and divalent cations using the numbers listed in Tables S1 and S2. The attempted concentrations were 100, 250, 500, 1000 mM for movalent and 25, 50 and 100 mM for divalent ions.

Ion	concentration (mM)			
	monovalent			
	100	250	500	1000
Li^+	98	234	470	938
Na^+	122	258	492	971
K^+	125	258	487	965
Cs^+	123	259	493	963
	divalent			
	25	50	100	
Mg^{2+}	31	52	118	
Ca^{2+}	28	49	116	
Sr^{2+}	23	44	110	
Ba^{2+}	33	53	119	

Table S4: Force field parameters for the metal cations used in this work. The charge and the Lennard-Jones parameters (σ and ϵ) are listed. Values were take from Refs.[S21] λ_σ^{iR} is the optimized scaling factor for the ion–phosphate oxygen interaction as described in Ref.[S22]

Ion	Charge (e)	σ (nm)	ϵ (kJ mol^{-1})	Charge density (e/nm^3)	λ_σ^{iR}
Li^+	+1.0	0.152	0.604	67.98	1.0
Na^+	+1.0	0.261	0.115	13.43	1.0
K^+	+1.0	0.299	0.604	8.93	1.0
Cs^+	+1.0	0.355	0.604	5.34	1.0
Mg^{2+}	+2.0	0.162	0.604	112.30	1.085
Ca^{2+}	+2.0	0.226	2.339	41.36	1.027
Sr^{2+}	+2.0	0.282	1.057	21.29	1.0
Ba^{2+}	+2.0	0.355	0.277	10.67	1.0

Table S5: Change in twist ΔTw (deg/bp) for monovalent cations from magnetic tweezer experiments.

Conc. (mM)	Li^+	Na^+	K^+	Rb^+	Cs^+
50	0.057 ± 0.008	-0.122 ± 0.016	-0.051 ± 0.019	0.001 ± 0.012	0.075 ± 0.020
100	0.104 ± 0.010	-0.080 ± 0.020	0	0.042 ± 0.011	0.118 ± 0.018
250	0.196 ± 0.010	-0.028 ± 0.017	0.057 ± 0.010	0.106 ± 0.012	0.191 ± 0.018
500	0.300 ± 0.015	0.050 ± 0.014	0.133 ± 0.010	0.190 ± 0.009	0.266 ± 0.026
1000	0.473 ± 0.023	0.149 ± 0.012	0.227 ± 0.015	0.299 ± 0.014	0.361 ± 0.018

Table S6: Fitting parameters for the experimental change in twist of monovalent cations, and their 95% lower- (LC) and upper confidence (UC) intervals.

	Ion	Parameter	Value	95% LC	95% UC	red. χ^2
$\Delta \text{Tw} = A(c)^B + C$	Li^+	A	-0.032	-0.078	0.015	0.102
		B	0.010	0.000	0.019	
		C	0.574	0.441	0.706	
	Na^+	A	-0.200	-0.296	-0.104	0.119
		B	0.012	-0.011	0.035	
		C	0.491	0.241	0.742	
	K^+	A	-0.146	-0.347	0.056	0.373
		B	0.020	-0.043	0.083	
		C	0.425	0.033	0.818	
	Rb^+	A	-0.088	-0.161	-0.016	0.104
		B	0.014	-0.005	0.033	
		C	0.483	0.308	0.659	
	Cs^+	A	-0.073	-0.099	-0.046	0.002
		B	0.036	0.025	0.047	
		C	0.359	0.323	0.395	
		TOTAL				0.699
$\Delta \text{Tw} = A + B\sqrt{c}$	Li^+	A	-0.060	-0.077	-0.044	0.257
		B	0.016	0.015	0.018	
	Na^+	A	-0.197	-0.212	-0.183	0.080
		B	0.011	0.010	0.012	
	K^+	A	-0.114	-0.137	-0.090	0.328
		B	0.011	0.010	0.012	
	Rb^+	A	-0.082	-0.092	-0.072	0.075
		B	0.012	0.012	0.013	
	Cs^+	A	0.002	-0.021	0.025	0.148
		B	0.012	0.010	0.013	
		TOTAL				0.888

Table S7: Change in twist ΔTw (deg/bp) for divalent cations from magnetic tweezer experiments.

Conc. (mM)	Mg^{2+}	Ca^{2+}	Sr^{2+}	Ba^{2+}
1	0.128 ± 0.018	0.281 ± 0.019	0.138 ± 0.022	-0.071 ± 0.020
2	0.170 ± 0.014	0.318 ± 0.020	0.155 ± 0.019	-0.058 ± 0.017
5	0.191 ± 0.008	0.362 ± 0.019	0.179 ± 0.017	-0.036 ± 0.012
10	0.206 ± 0.010	0.367 ± 0.025	0.182 ± 0.022	-0.028 ± 0.019
25	0.231 ± 0.014	0.387 ± 0.023	0.199 ± 0.020	-0.019 ± 0.027
50	0.246 ± 0.010	0.416 ± 0.022	0.201 ± 0.028	-0.009 ± 0.022
100	0.272 ± 0.013	0.427 ± 0.024	0.232 ± 0.019	0.009 ± 0.025

Table S8: Fitting parameters for the experimental change in twist of divalent cations, and their 95% lower- (LC) and upper confidence (UC) intervals.

	Ion	Parameter	Value	95% LC	95% UC	red. χ^2
$\Delta\mathrm{Tw} = A(c)^B + C$	Mg^{2+}	A	1.000			
		B	0.026	0.021	0.031	
		C	-0.859	-0.874	-0.844	0.402
	Ca^{2+}	A	1.000			
		B	0.028	0.213	0.320	
		C	-0.703	-0.724	-0.681	0.385
	Sr^{2+}	A	1.000			
		B	0.017	0.131	0.2161	
		C	-0.858	-0.871	-0.845	0.116
	Ba^{2+}	A	1.000			
		B	0.015	0.133	0.181	
		C	-1.068	-1.075	-1.060	0.071
		TOTAL				0.976
$\Delta\mathrm{Tw} = A + B\ln(c)$	Mg^{2+}	A	0.145	0.133	0.157	0.296
		B	0.027	0.022	0.031	
	Ca^{2+}	A	0.295	0.275	0.315	0.339
		B	0.031	0.023	0.038	
	Sr^{2+}	A	0.142	0.131	0.153	0.106
		B	0.018	0.014	0.023	
	Ba^{2+}	A	-0.067	-0.074	-0.059	0.058
		B	0.016	0.013	0.019	
		TOTAL				0.799

References

(S1) Darden, T.; York, D.; Pedersen, L. Particle mesh Ewald: An N·log(N) method for Ewald sums in large systems. *J. Chem. Phys.* **1993**, *98*, 10089–10092.

(S2) Hess, B.; Bekker, H.; Berendsen, H. J. C.; Fraaije, J. G. E. M. LINCS: A linear constraint solver for molecular simulations. *J. Comput. Chem.* **1997**, *18*, 1463–1472.

(S3) Jorgensen, W. L.; Chandrasekhar, J.; Madura, J. D.; Impey, R. W.; Klein, M. L. Comparison of simple potential functions for simulating liquid water. *J. Chem. Phys.* **1983**, *79*, 926–935.

(S4) Parrinello, M.; Rahman, A. Polymorphic transitions in single crystals: A new molecular dynamics method. *J. Appl. Phys.* **1981**, *52*, 7182–7190.

(S5) Bussi, G.; Donadio, D.; Parrinello, M. Canonical sampling through velocity rescaling. *J. Chem. Phys.* **2007**, *126*, 014101.

(S6) Lu, X.-J.; Olson, W. K. 3DNA: a versatile, integrated software system for the analysis, rebuilding and visualization of three-dimensional nucleic-acid structures. *Nat. Protoc.* **2008**, *3*, 1213.

(S7) Kumar, R.; Grubmüller, H. do_x3dna: a tool to analyze structural fluctuations of dsDNA or dsRNA from molecular dynamics simulations. *Bioinformatics* **2015**, *31*, 2583–2585.

(S8) Marin-Gonzalez, A.; Vilhena, J. G.; Moreno-Herrero, F.; Perez, R. DNA Crookedness Regulates DNA Mechanical Properties at Short Length Scales. *Phys. Rev. Lett.* **2019**, *122*, 048102.

(S9) Lavery, R.; Maddocks, J. H.; Pasi, M.; Zakrzewska, K. Analyzing ion distributions around DNA. *Nucleic Acids Res.* **2014**, *42*, 8138–8149.

(S10) Briones, R.; Blau, C.; Kutzner, C.; de Groot, B. L.; Aponte-Santamaría, C. GROmaρs: A GROMACS-Based Toolset to Analyze Density Maps Derived from Molecular Dynamics Simulations. *Biophys. J.* **2019**, *116*, 4 – 11.

(S11) DeLano, W. L., et al. Pymol: An open-source molecular graphics tool. *CCP4 Newsl. Protein Crystallogr.* **2002**, *40*, 82–92.

(S12) Kriegel, F.; Matek, C.; Dršata, T.; Kulenkampff, K.; Tschirpke, S.; Zacharias, M.; Lankaš, F.; Lipfert, J. The temperature dependence of the helical twist of DNA. *Nucleic Acids Res.* **2018**, *46*, 7998–8009.

(S13) Olson, W. K.; Bansal, M.; Burley, S. K.; Dickerson, R. E.; Gerstein, M.; Harvey, S. C.; Heinemann, U.; Lu, X.-J.; Neidle, S.; Shakked, Z.; Sklenar, H.; Suzuki, M.; Tung, C.-S.; Westhof, E.; Wolberger, C.; Berman, H. M. A standard reference frame for the description of nucleic acid base-pair geometry. *J. Mol. Biol.* **2001**, *313*, 229–237.

(S14) Dršata, T.; Réblová, K.; Beššeová, I.; Šponer, J.; Lankaš, F. rRNA C-Loops: Mechanical Properties of a Recurrent Structural Motif. *J. Chem. Theory Comput.* **2017**, *13*, 3359–3371.

(S15) Dans, P. D.; Pérez, A.; Faustino, I.; Lavery, R.; Orozco, M. Exploring polymorphisms in B-DNA helical conformations. *Nucleic Acids Res.* **2012**, *40*, 10668–10678.

(S16) Dans, P. D.; Faustino, I.; Battistini, F.; Zakrzewska, K.; Lavery, R.; Orozco, M. Unraveling the sequence-dependent polymorphic behavior of d(CpG) steps in B-DNA. *Nucleic Acids Res.* **2014**, *42*, 11304–11320.

(S17) Zgarbová, M.; Jurečka, P.; Lankaš, F.; Cheatham, T. E.; Šponer, J.; Otyepka, M. Influence of BII Backbone Substates on DNA Twist: A Unified View and Comparison of Simulation and Experiment for All 136 Distinct Tetranucleotide Sequences. *J. Chem. Inf. Model.* **2017**, *57*, 275–287.

(S18) Bai, Y.; Greenfeld, M.; Travers, K. J.; Chu, V. B.; Lipfert, J.; Doniach, S.; Herschlag, D. Quantitative and Comprehensive Decomposition of the Ion Atmosphere around Nucleic Acids. *J. Am. Chem. Soc.* **2007**, *129*, 14981–14988.

(S19) Lipfert, J.; Doniach, S.; Das, R.; Herschlag, D. Understanding Nucleic Acid–Ion Interactions. *Annu. Rev. Biochem.* **2014**, *83*, 813–841.

(S20) Howerton, S. B.; Sines, C. C.; VanDerveer, D.; Williams, L. D. Locating monovalent cations in the grooves of B-DNA. *Biochemistry* **2001**, *40*, 10023–10031.

(S21) Mamatkulov, S.; Schwierz, N. Force fields for monovalent and divalent metal cations in TIP3P water based on thermodynamic and kinetic properties. *J. Chem. Phys.* **2018**, *148*, 074504.

(S22) Cruz-León, S.; Grotz, K. K.; Schwierz, N. Extended magnesium and calcium force field parameters for accurate ion–nucleic acid interactions in biomolecular simulations. *J. Chem. Phys.* **2021**, *154*, 171102.

(S23) Berendsen, H.; Grigera, J.; Straatsma, T. The missing term in effective pair potentials. *J. Chem. Phys.* **1987**, *91*, 6269–6271.
